# A multimodal generative model for structured and unstructured electronic health records

**DOI:** 10.1038/s44401-026-00095-y

**Published:** 2026-06-15

**Authors:** Sonish Sivarajkumar, Hang Zhang, Yuelyu Ji, Maneesh Bilalpur, Xizhi Wu, Chenyu Li, Min Gu Kwak, Shyam Visweswaran, Yanshan Wang

**Affiliations:** 1 Intelligent Systems Program, University of Pittsburgh, Pittsburgh, PA USA; 2 Department of Health Information Management, University of Pittsburgh, Pittsburgh, PA USA; 3 Department of Biomedical Informatics, University of Pittsburgh, Pittsburgh, PA USA; 4 Clinical and Translational Science Institute, University of Pittsburgh, Pittsburgh, PA USA; 5 Hillman Cancer Center, University of Pittsburgh Medical Center, Pittsburgh, PA USA

**Keywords:** Computational biology and bioinformatics, Diseases, Health care, Mathematics and computing, Medical research

## Abstract

Electronic health records (EHRs) are rich clinical data sources but complex repositories of patient data, spanning structured elements (demographics, vitals, lab results, codes), unstructured clinical notes and other modalities of data. Harnessing this heterogeneity for AI-driven clinical insight remains challenging: most current approaches either serialize numeric EHR data into text, risking loss of temporal and quantitative detail, or learn patient embeddings from structured data alone without generative capability. We present Generative Deep Patient (GDP), a multimodal generative model trained on Medical Information Mart for Intensive Care (MIMIC)-IV that jointly models structured EHR time-series and unstructured clinical texts. GDP encodes structured EHR events via a Convolutional Neural Network (CNN)-Transformer encoder and fuses them with clinical text representations through cross-modal attention into a Large Language Model Meta AI (LLaMA)-based generative decoder. GDP is trained using a combination of generative pretraining and auxiliary temporal objectives, followed by multi-task fine-tuning for clinical prediction and narrative generation. Evaluated on the MIMIC-IV dataset, GDP achieved strong predictive performance for heart failure (Area Under the Receiver Operating Characteristic [AUROC] = 0.923), type 2 diabetes (AUROC = 0.817), and 30-day readmission (AUROC = 0.627). In narrative generation, GDP produced clinically coherent discharge summaries with Recall-Oriented Understudy for Gisting Evaluation (ROUGE)-L = 0.135 and Bidirectional Encoder Representations from Transformers Score (BERTScore)-F1 = 0.545. Human evaluation demonstrated high faithfulness, fluency, and clinical utility. These findings demonstrate that unified multimodal generative modeling of structured EHR and clinical text is feasible and yields competitive performance on multiple downstream tasks in MIMIC-IV, informing future EHR-scale multimodal model development.

## Introduction

Electronic health records (EHRs), which compile longitudinal histories of patients from a variety of data modalities, have become a vast and transformative resource for clinical tasks Structured or codified data in EHRs (e.g., demographics, diagnoses, procedures, vital signs, laboratory test results, and medications) and unstructured or free-text narratives together capture a comprehensive clinical picture. Leveraging this rich data for AI-driven insights poses significant challenges: the data are high-dimensional, longitudinal, irregularly sampled, heterogeneous, and often contain noise and missing values^1^. Traditional machine learning approaches struggle with the sheer volume and complexity of EHRs. A critical step is learning meaningful patient representations from these multimodal sources, which can enable accurate predictions of outcomes such as readmission, or new diagnoses^2^.

Recent advances in deep learning, large language models (LLMs), and generative modeling have spurred their application to healthcare^3^. Deep models, neural networks with multiple hidden layers, learn hierarchical representations at different levels of abstraction. Foundation models are large-scale models trained on broad, diverse data that can be adapted to many downstream tasks through fine-tuning or prompting. Generative models learn the underlying data distribution and can produce new, realistic data. These paradigms are not mutually exclusive: modern systems such as GPT-4 are simultaneously deep, generative, and foundational. In the EHR domain, these paradigms motivate models that can both learn patient representations and generate clinically useful outputs.

In the context of EHRs, researchers have explored two main directions: (1) Clinical language models are trained on clinical free text narratives to understand and generate clinically relevant language (e.g., Clinical Language Models, or CLaMs)^4,5^, and (2) Foundation models learn patient representations from structured EHR timelines and can be adapted (via fine-tuning or prompting) to perform specific clinical tasks (e.g., Foundation models for Electronic Medical Records, or FEMRs)^6,7^. While CLaMs treat input as tokens, structured EHR data is inherently numerical, so transforming structured EHR into tokens and then encoding them back into an LLM decoder might not be the most effective approach. Moreover, converting numerical and time-series EHR elements into text tokens for LLMs can degrade numeric precision and temporal context—factors that are often critical for patient safety and risk stratification. FEMRs, on the other hand, produce dense patient embeddings from a patient’s entire records, which can feed downstream predictive models. Studies have shown that such EHR-specific foundation models can improve predictive accuracy and require fewer labeled examples than traditional methods. For instance, self-supervised models trained on large EHR datasets have achieved state-of-the-art results on clinical risk prediction while maintaining robustness to shifts in data distribution^8^. At the same time, general-purpose LLMs like GPT have demonstrated impressive language understanding and medical knowledge, but often underperform domain-specific models, especially on structured prediction tasks. Specialized biomedical language models, such as Bidirectional Encoder Representations from Transformers for Biomedicine (BioBERT)^9^ and GatorTron^10^, have been developed to better capture medical language compared to general language models. However, they lack the generative capabilities of LLMs and are not optimized for question answering tasks.

One recently proposed approach to bridge structured EHR data with LLMs is to serialize patient records into textual form that LLMs can interpret^11,12^. In this paradigm, as in CLaMs, all structured elements (e.g., codes, time-stamped events) are converted into a formatted text (such as a lengthy Markdown document) with human-readable descriptions. An LLM (or an LLM-based embedding model) processes this text to yield a patient embedding. This strategy leverages LLMs’ broad knowledge and avoids needing a proprietary EHR corpus for initial training. However, this serialized-text approach has several limitations. First, it converts numerical variables into text, and the LLM encodes it into numerical vectors, during which the data loses various contexts and details^13^. Second, it relies on a hand-crafted schema for representing structured data as text, which may introduce biases or omit subtle information^14^. Model performance can be sensitive to the exact formatting and phrasing of the serialized record, raising reproducibility concerns. Third, processing long textual records pushes against LLM context length limits - important historical data may be truncated, especially for patients with lengthy records^15^. Perhaps most critically, when the LLM’s output is used only as features for a separate classifier (for example, a logistic regression on the LLM embedding), we lose the LLM’s native ability to perform reasoning or generation in the final step of the task. In other words, the pipeline forfeits the zero-shot and conversational capabilities of the LLM at the point of making clinical predictions. These observations motivate a unified multimodal framework that treats structured EHR and clinical text as complementary inputs—each encoded in its native form—rather than flattening all signals into text.

An alternative paradigm, supported by extensive prior work, is multimodal learning: designing models that explicitly integrate each data modality in its native form^16^. Instead of flattening everything into text, a multimodal architecture can use appropriate subnetworks for structured numerical data, time-series signals, and text, and then fuse these representations. By preserving each modality’s structure, such models can capture patterns that might be lost in text translation. Multimodal fusion approaches have shown improved performance on clinical predictions by combining complementary information sources. For example, earlier deep learning systems combined coded data and clinical notes via separate encoders (e.g., recurrent networks or convolutional neural networks for sequences of codes and transformers^17^ for text) and achieved higher accuracy than single-modality models. This suggests that a holistic model, ingesting the full spectrum of EHR data, could learn more robust patient representations and yield better predictions than models that treat the record as only text or only codes.

Other works have explored multimodal modeling approaches that integrate heterogeneous clinical data sources. For example, Liu et al.^20^ developed a multimodal large language model combining clinical text, medical imaging, and genomic data to support pandemic response through cross-modal reasoning for infectious disease surveillance. Similarly, Gu et al.^21^ introduced a large-scale cardiac foundation model trained on 1.7 million individuals that integrates electrocardiogram (ECG) waveforms, imaging, and clinical measurements across heterogeneous devices and clinical settings. In parallel, research on clinical text generation has explored retrieval-augmented architectures. Liu et al.^22^ proposed Retrieve, Reason, and Refine (Re3), which generates patient-facing discharge instructions by retrieving similar historical cases from a knowledge base and iteratively refining generated outputs. While these studies demonstrate the potential of multimodal learning and generative modeling in healthcare, they focus on specialized modalities such as imaging, waveforms, genomic data, or retrieval-augmented pipelines for patient-facing instructions. In contrast, our work addresses the complementary challenge of jointly modeling routine structured EHR events, such as laboratory tests, medications, diagnoses, and procedures, together with unstructured clinical narratives within a unified generative framework. Specifically, we develop Generative Deep Patient (GDP), which integrates structured EHR timelines and clinical text within a single LLM-style decoder capable of both predictive tasks and long-form clinical narrative generation, and is trained using a unified framework that combines masked feature prediction, next-event prediction, and discharge summary generation on the same patient cohort.

**Fig. 1:**
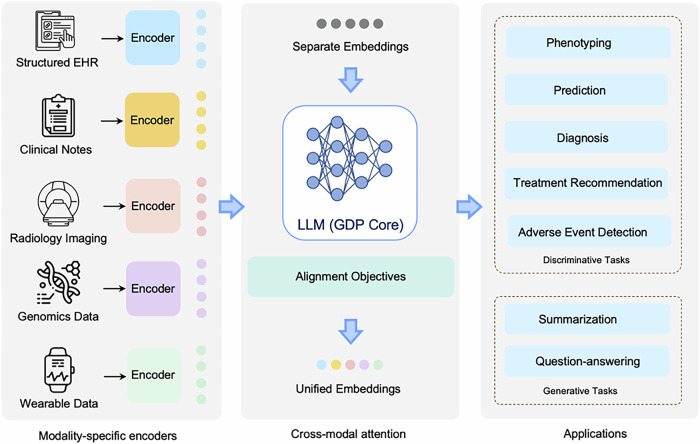
A multimodal architecture for generative clinical AI models. In this paper, we focus on combining the first two modalities, i.e., structured EHRs and unstructured free-text EHRs. Discriminative tasks—directly predict specific outcomes or labels from patient data (e.g., diagnosing heart failure or estimating readmission risk). Generative tasks - produce new clinical data, specifically text in this study, (e.g., discharge summaries or progress notes).

GDP architecture (Fig. 1) employs dedicated encoders for structured EHR time-series data and for unstructured text, and then fuses their outputs into a unified latent representation using an LLM backbone with cross-modal attention^18^. This design allows the model to absorb the rich temporal patterns of structured data as well as the semantic context of clinical text. Uniquely, we explore two variants of the LLM backbone: one initialized with an instruction-tuned model (GDP-Instruct) and one with a standard pre-trained model (GDP-Base)^19^.

GDP demonstrates effective system-level integration of established components like convolutional-transformer encoders for structured EHR, pretrained clinical language models for text, and cross-modal attention fusion within a generative decoder, into a unified framework optimized for routine EHR data. Several aspects distinguish GDP’s design from prior work:

1. Unified generative architecture for EHR: Unlike models that use separate encoders for multimodal fusion but employ discriminative heads for each task, GDP integrates both structured EHR events and clinical text into a single LLM-style generative decoder that supports both classification and free-text narrative generation from the same patient representation.

2. Joint training on EHR structure and text: GDP combines self-supervised objectives over structured EHR (masked feature prediction, next-step prediction) with generative pre-training on discharge summaries in a unified training recipe, enabling the model to learn from both modalities simultaneously on the same patient cohort.

3. Empirical demonstration on Medical Information Mart for Intensive Care (MIMIC)-IV data: We provide systematic evaluation showing that this integrated approach achieves competitive or superior performance compared to strong unimodal and multimodal baselines across clinically meaningful tasks.

We position GDP as a reference implementation demonstrating that routine EHR structure and clinical text can be effectively unified in a generative framework, which may inform future EHR-scale model development. We evaluate GDP on multiple clinically meaningful downstream tasks, including three binary diagnosis prediction tasks (heart failure, type 2 diabetes, and 30-day readmission) and one generative task (discharge summary generation). While these tasks demonstrate the model’s adaptability to both discriminative and generative objectives, they represent a subset of potential clinical applications.

## Results

GDP is designed to perform well in both discriminative and generative tasks. A discriminative task is one where the model learns to predict a specific outcome or label from patient data. For example, predicting HF or assessing the readmission of a patient after hospital discharge. Discriminative models directly approximate the conditional probability P(label∣data) and are evaluated by classification metrics (e.g., AUROC, AUPRC, F1). In contrast, a generative task is one where the model learns to produce new, realistic clinical text based on the same patient data, such as generating a “Brief Hospital Course” summary. Generative models approximate the joint or conditional probability of the text given the data, P(text∣data), and are evaluated by language-generation metrics (e.g., ROUGE, BLEU, BERTScore) or human assessment of fluency and faithfulness.

We evaluated GDP in three phases:

1. Clinical prediction tasks (discriminative): We selected three key outcomes—heart failure (HF), type 2 diabetes mellitus (T2DM), and 30-day readmission—because they represent high-impact conditions where early identification can change management (e.g., adjusting diuretics for HF, tighter glycemic control for T2DM, care-transition planning to reduce readmissions).
2. Clinical narrative generation (generative): We evaluated how accurately the model can produce the “Brief Hospital Course” section of discharge summaries, since concise, accurate hand-offs are critical for continuity of care.
3. Ablation and subjective evaluation: We performed ablation to isolate the contributions of auxiliary objectives (masked feature prediction using label “[MFP]” and next time-step prediction using label “[NTP]”) and “instruction tuning,” as well as a blinded human rating to assess faithfulness and utility (Supplementary Material Section 4 and 5).

**Fig. 2:**
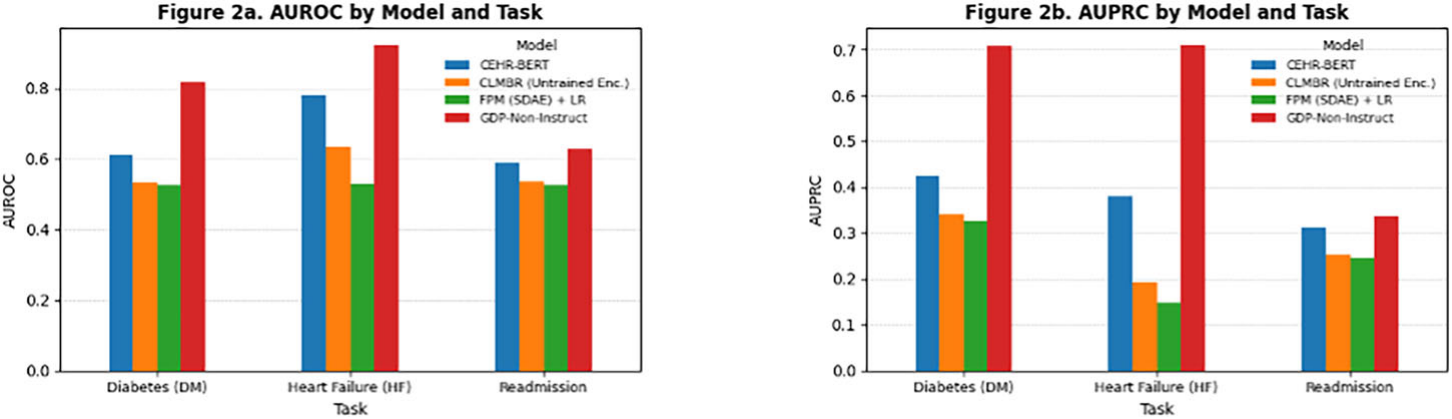
Discriminative performance across clinical prediction tasks. **a** and **b** shows Area Under the Receiver Operating Characteristic curve (AUROC) and Area Under the Precision-Recall Curve (AUPRC), respectively, for T2DM, HF, and 30-day readmission, comparing GDP and three baseline models.

**Fig. 3:**
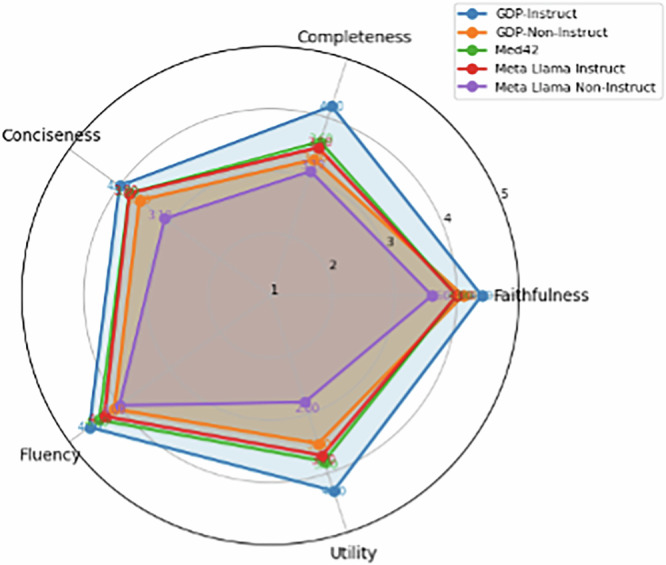
Human evaluation radar chart. Mean Likert ratings (1–5 scale) for faithfulness, completeness, conciseness, fluency, and clinical utility of discharge-summary generation outputs, averaged over two expert evaluators (*n* = 200 summaries per model).

Below, we first present results on the discriminative tasks (Table 1, Fig. 2), then on narrative generation (Tables 2, 3, Fig. 3), followed by ablation analyses and qualitative chat demonstrations.

**Table 1:** Discriminative performance metrics on EHR prediction tasks

Model	Task	F1-Score	Precision	Recall	Accuracy
CEHR-BERT	ICD428 (HF)	0.2554	0.55	0.17	0.87
	ICD250 (T2DM)	0.1323	0.62	0.07	0.70
	Readmission	0.0312	0.61	0.03	0.77
FPM (SDAE) + LR	ICD428 (HF)	0.2317	0.13	0.79	0.13
	ICD250 (T2DM)	0.4657	0.30	0.70	0.30
	Readmission	0.430	0.35	0.55	0.77
CLMBR	ICD428 (HF)	0.2854	0.18	0.66	0.57
	ICD250 (T2DM)	0.3956	0.33	0.50	0.53
	Readmission	0.3336	0.25	0.52	0.52
GDP-Non-Instruct	ICD428 (HF)	0.6020	0.47	0.84	0.85
	ICD250 (T2DM)	0.6218	0.55	0.72	0.74
	Readmission	0.3967	0.30	0.57	0.60

F1-score, precision, recall, and accuracy for GDP variants and baseline models (CEHR-BERT, FPM + LR, CLMBR) on HF (ICD-9 428.x), T2DM (ICD-9 250.x), and 30-day readmission.

**Table 2:** Automated generation metrics for discharge summary

Model	ROUGE-1 (F)	ROUGE-2 (F)	ROUGE-L (F)	BLEU-4	BERTScore-F1
Meta Llama-Non-Instruct	0.11	0.01	0.08	0.01	0.44
Meta Llama Instruct	0.12	0.01	0.08	0.01	0.46
Med42	0.17	0.03	0.11	0.01	0.5
GDP-Non-Instruct	0.2	0.04	0.13	0.01	0.54
GDP-Instruct	0.21	0.05	0.14	0.02	0.55

ROUGE-1, ROUGE-2, ROUGE-L F1-scores, BLEU-4, and BERTScore-F1 for baseline LLaMA models (instruct/non-instruct), Med42, and both GDP variants.

**Table 3:** Mean human evaluation scores (1–5 Likert) for generated discharge summaries

Model	Faithfulness	Completeness	Conciseness	Fluency	Utility
GDP-Instruct	4.4	4.2	4.0	4.6	4.3
GDP-Non-Instruct	4.1	3.3	3.6	4.1	3.5
Med42	4.0	3.6	3.8	4.4	3.8
Meta Llama Non-Instruct	3.6	3.1	3.1	4.0	2.8
Meta Llama Instruct	4.0	3.5	3.8	4.3	3.7

Ratings for faithfulness, completeness, conciseness, fluency, and clinical utility averaged over two expert annotators; inter-annotator agreement *κ* = 0.68.

### Cohort characteristics

The MIMIC-IV cohort used in this study comprised admissions to Beth Israel Deaconess Medical Center between 2008 and 2019. The median patient age was 65 years (IQR: 52–77), with 54.3% male patients. Median length of stay was 4.2 days (IQR: 2.1–8.6). The three most common primary diagnosis categories were circulatory system diseases (21.4%), respiratory diseases (12.8%), and injury/poisoning (11.2%). These statistics contextualize the clinical complexity and diversity of the prediction tasks.

### Predictive performance on clinical tasks

We evaluated the fine-tuned GDP models on three key prediction tasks derived from the hospital EHR data: identification of two prevalent chronic conditions (HF and T2DM) based on data available early in admission, and 30-day hospital readmission. We compared GDP against three existing approaches: (a) CEHR-BERT, a Transformer-based EHR sequential model^23^, (b) FPM (SDAE)^8^, a stacked denoising autoencoder on EHR data with logistic regression based on the deep patient model^24^, and (c) CLMBR, a recent self-supervised EHR foundation model (141 M parameters) that autoregressively predicts the next code in a patient timeline^6^. We selected these approaches to span different modeling strategies—Transformer-only, autoencoder-plus-classifier, and autoregressive EHR models, that GDP’s multimodal fusion and pretraining approach could be fairly compared against diverse, high-performing alternatives. Figure 2 illustrates the AUROC and AUPRC curves for the models on each task.

#### Key terms

- *Transformer—a deep learning architecture that uses self-attention to model relationships between events or tokens in a sequence*.
- *Autoencoder—a neural network that compresses data into a lower-dimensional representation (encoder) and reconstructs it (decoder), often used for feature learning*.
- *Classifier—a model component that assigns an input to a discrete label (e.g., presence of heart failure vs. not)*.
- *Autoregression—a modeling approach where the next element in a sequence is predicted based on its past values*.

Overall, the GDP models achieved the highest discriminative performance. For HF prediction, GDP-Non-Instruct obtained an AUROC of 0.923 (95% CI ~ 0.91–0.93) and AUPRC of 0.71, substantially outperforming the best baseline (CEHR-BERT, AUROC 0.779, AUPRC 0.38) and the CLMBR model (AUROC 0.64, AUPRC 0.19). Its F1-score was 0.60, far higher than the baseline CEHR-BERT’s 0.25 (the latter struggled with very low recall of 0.17 for HF). T2DM prediction showed a similar pattern: GDP-Non-Instruct reached AUROC 0.817 and AUPRC 0.707, versus 0.610 and 0.424 for CEHR-BERT. The simpler baselines (SDAE and CLMBR) performed near chance on HF and T2DM (AUROCs ~0.53–0.63). On the more challenging 30-day readmission task, all models had lower scores, reflecting the inherent difficulty of predicting readmissions. GDP-Non-Instruct again led with AUROC 0.627 and AUPRC 0.335, compared to 0.589 and 0.311 for the best baseline (CEHR-BERT). Notably, CEHR-BERT’s F1 on readmission was only 0.03 (it almost never predicted the positive class, yielding recall 1.6%), whereas GDP-Non-Instruct achieved F1 ≈ 0.40 by balancing precision and recall (~0.30 and 0.57, respectively). The differences between GDP-Non-Instruct and the best baseline (CEHR-BERT) were statistically significant in the HF and T2DM tasks. In readmission, the improvement was modest. These results demonstrate that GDP’s multimodal fusion and pretraining strategy conferred a sizable advantage over prior EHR-specific models in two of three evaluated clinical predictions. We attribute this to GDP’s ability to capture long-term temporal patterns (through its CNN–Transformer encoder and NTP objective) and to integrate information across notes and coded data. The instruction fine-tuning of the LLM, however, was not necessary (and perhaps slightly disadvantageous) for maximizing structured-task performance in this setting.

### Generative performance on clinical text

We next evaluated each model’s ability to generate specific sections of a discharge summary from structured EHR inputs. In this simulated setup, models receive a patient’s admission events and context and must produce the concise narrative clinicians write at discharge. We fine-tuned both GDP-Instruct and GDP-Non-Instruct on paired structured data and ground-truth summary text, and compared them against three existing models: (1) a 3B-parameter LLaMA-derived model in non-instruct (“Meta Llama Non-Instruct”) and instruct-tuned (“Meta Llama Instruct”) forms^25^, and (2) Med42, an 8B clinical LLM based on Llama-2 and instruction-tuned on medical data^26^. These three baselines were chosen to have a comprehensive comparison on non-instruct(base), instruct and a finetuned versions of LLMs.

We report standard automated language-generation metrics-ROUGE-1, ROUGE-2, and ROUGE-L F-scores (n-gram overlap measures), BLEU-4 (precision-oriented n-gram overlap), and BERTScore-F1 (semantic similarity)-for all model outputs (Table 2). Higher values indicate closer agreement with the reference clinician-written summaries.

GDP-Instruct demonstrated superior performance on every metric-ROUGE-1 of 0.215, ROUGE-2 of 0.048, and ROUGE-L of 0.135-demonstrating superior n-gram overlap with the reference summaries. Its BLEU-4 score (0.017) further indicates more accurate reproduction of multi-word sequences, and its BERTScore-F1 (0.545) confirms best semantic fidelity among the models. GDP-Non-Instruct ranked second, outperforming the much larger Med42 model (ROUGE-L = 0.105, BLEU-4 = 0.009, BERTScore = 0.500) despite having only 3 billion parameters. The instruct-tuned Meta Llama baseline showed only modest gains over its non-instruct version (ROUGE-L = 0.075 vs. 0.075, BERTScore = 0.460 vs. 0.435), underscoring the benefit of both instruction tuning and our EHR-focused pretraining.

A ROUGE-L F1 of 0.135 indicates that the model captures approximately 13.5% of the longest common subsequence between its output and the reference “Brief Hospital Course,” which may seem low by general NLP standards—but in clinical practice, even capturing key events (e.g., “IV antibiotics started,” “elevated troponin noted,” “discharged on beta-blocker”) can substantially reduce clinician documentation time. These quantitative results highlight that targeted pretraining on clinical data, combined with instruction optimization, yields high-quality, clinically faithful summaries without requiring enormous model scale.

In addition to automated metrics, we conducted a blind human evaluation of the generated summaries to assess their clinical usefulness and correctness, adhering to QUEST guidelines^27^ (Table 3). Two health informatics specialists reviewed a randomized sample of 200 summary outputs per model, rating each on five criteria, as defined by QUEST human evaluation framework^27^: Faithfulness, or how accurately the summary reflects the patient’s clinical facts; Completeness, meaning coverage of all key clinical events; Conciseness, which captures essential information without unnecessary detail; Fluency, referring to grammatical smoothness and readability; and Overall Clinical Utility, indicating how useful the summary would be for real-world decision-making. Ratings used a 1–5 Likert scale (5 = best), and evaluators were blinded to the model source. Inter-annotator agreement was substantial (overall Cohen’s κ = 0.68), with *κ* by criterion as follows: Faithfulness 0.62, Completeness 0.64, Conciseness 0.66, Fluency 0.75, and Utility 0.60. The scores suggest that GDP can generate high-quality data while maintaining predictive accuracy.

GDP-Instruct achieved the highest mean rating on four of five criteria—most notably Fluency (4.6) and Faithfulness (4.4)—and matched the top Conciseness score (4.0). It outperformed the next-best model (Meta Llama Instruct) by 0.5 points on Completeness and Utility. The evaluators noted that GDP-Instruct outputs “generally stick to documented facts without hallucinating,” whereas other models occasionally omitted or misstated key events. GDP-Non-Instruct performed reasonably but lagged behind GDP-Instruct across all dimensions, particularly in Utility (3.5), reflecting less coherent and actionable narratives without instruction tuning. Figure 3 presents these results in a radar chart, illustrating GDP-Instruct’s uniformly stronger performance across clinical evaluation axes. These findings confirm that instruction tuning on EHR-derived tasks substantially enhances both the factual reliability and practical value of generated discharge summaries.

### Qualitative analysis

While automatic metrics provide quantitative benchmarks, they have known limitations in capturing clinical utility, factual accuracy, and narrative coherence. Table 4 presents qualitative examples comparing reference and GDP-Instruct-generated “Brief Hospital Course” summaries for three representative cases: (1) a cardiac case with heart failure exacerbation, (2) an endocrine case with diabetic ketoacidosis, and (3) a surgical case with post-operative complications. GDP-Instruct successfully captures key clinical events and temporal progression in most cases, maintaining appropriate clinical terminology and structure. Occasional omissions of secondary diagnoses and procedural details are observed. Generated summaries tend to be slightly more concise than reference summaries.

**Table 4:** Qualitative examples of discharge summary generation

Case	Reference “Brief Hospital Course”	GDP-Instruct Generated Summary	Annotations
1. Cardiac (Heart Failure Exacerbation)	68-year-old male with HFrEF presented with worsening dyspnea and edema. Found to have acute decompensated heart failure. Started on IV furosemide with good urine output, transitioned to oral torsemide on hospital day 3. Echocardiogram showed EF 30% unchanged from prior. Discharged home stable on baseline GDMT.	68-year-old male with HFrEF admitted for acute decompensated heart failure with dyspnea and edema. Treated with IV furosemide and diuresed well. Transitioned to oral torsemide. Echocardiogram revealed EF 30%. Discharged stable on guideline-directed medical therapy.	Factual Accuracy: Maintained. Temporal Ordering: Correct IV → oral transition. Omissions: Did not mention hospital day 3 transition.
2. Endocrine (Diabetic Ketoacidosis)	24-year-old female with type 1 diabetes admitted for DKA triggered by viral URI. Presented with nausea, vomiting, glucose 520 mg/dL, high anion gap. Managed with insulin drip and aggressive IV fluids. Gap closed within 16 hours. Transitioned to glargine and lispro. Educated on sick day rules and discharged.	24-year-old female with T1DM admitted for DKA secondary to viral URI. Glucose 520 with high anion gap. Managed with IV fluids and insulin drip. Anion gap closed. Transitioned to subcutaneous glargine and lispro. Discharged after diabetes education.	Factual Accuracy: Accurate clinical parameters. Clinical Relevance: Strong summary of critical management steps. Omissions: Did not specify 16-hour gap closure.
3. Surgical (Post-op Complications)	55-year-old male underwent elective laparoscopic cholecystectomy. Post-op course complicated by low-grade fever and mild RUQ pain on POD 1. Ultrasound negative for biliary leak or collection. Fever resolved spontaneously. Advanced diet tolerated. Discharged POD 2 with oral analgesics.	55-year-old male s/p elective lap chole. Course complicated by fever and RUQ pain on POD 1. Ultrasound showed no biliary leak. Fever resolved. Tolerated diet and discharged POD 2 with pain medications.	Factual Accuracy: Captures complication clearly. Temporal Ordering: Clear POD 1 → POD 2 progression. Fluency: Uses appropriate clinical shorthand.

Side-by-side comparisons of reference vs GDP-Instruct-generated “Brief Hospital Course” for three representative cases, with annotations highlighting factual accuracy, temporal ordering, clinical relevance, and omissions.

### Ablation summary

Ablation analysis (Supplementary Table S2) demonstrates the contribution of each pre-training objective. Removing NTP during pretraining resulted in AUROC decreases of approximately 3 points for HF (0.923 → 0.893) and 2 points for T2DM (0.817 → 0.795). Removing both MFP and NTP yielded the largest performance decrease (HF AUROC: 0.923 → 0.870; T2DM AUROC: 0.817 → 0.780). These results confirm that the auxiliary self-supervised objectives contribute meaningfully to downstream discriminative performance, with NTP providing the larger individual contribution by encouraging the encoder to model temporal disease progression

## Discussion

In this work, we introduced GDP, a multimodal generative model that jointly processes structured EHR data and unstructured clinical text within a unified architecture for both predictive and generative tasks. While recent models in healthcare have demonstrated capabilities with clinical text and specialized modalities^28,29^, the integration of routine structured EHR time-series with clinical narratives in a single generative framework remains underexplored. GDP addresses this gap by demonstrating that such integration is feasible and yields competitive performance on the MIMIC-IV dataset.

The results demonstrate that a single model can indeed perform well on both structured outcome prediction and generative tasks. In GDP-Instruct, instruct LLM backbone translated to superior performance in the summary generation and a smoother conversational ability. The model could leverage its instruction-following training to organize information from the EHR into a fluent summaries effectively. On the other hand, GDP-Non-Instruct’s backbone remained closer to a standard language model that optimizes next-token prediction on general text. After fine-tuning on our supervised tasks, it appeared to retain a sharper focus on the discriminative signals in the data, yielding higher AUROC/AUPRC. One hypothesis is that the instruction-tuned model had been predisposed to prioritize natural language coherence and high-level reasoning, which might introduce a form of regularization (or even a slight distraction) when it is repurposed for strict classification objectives. The non-instruct model, being more “raw”, might more readily memorize and exploit subtle statistical patterns in the structured data during fine-tuning, hence its edge in predictive performance. This echoes observations in other multimodal domains where instruction-tuned vision-language models trade some accuracy for better alignment and usability^30^.

GDP’s design philosophy prioritizes practical integration of proven components over algorithmic novelty. The CNN-Transformer encoders, BERT-style text encoders, cross-attention fusion, and self-supervised objectives we employ have each been validated individually in prior work. Our contribution lies in demonstrating that these components can be systematically combined into a unified generative model for EHR data and that this integration yields strong empirical performance on multiple downstream tasks. We view GDP as a building block for future research rather than an endpoint, providing a baseline architecture that others can extend with more sophisticated fusion mechanisms, retrieval augmentation, or multi-task learning frameworks. The three auxiliary objectives we employ, masked prediction, next-step forecasting, and text generation, are borrowed from successful paradigms in other domains and applied in a straightforward manner to multimodal EHR. Our empirical results suggest that this combination is effective for MIMIC-IV. Developing principled frameworks for multimodal pre-training on heterogeneous clinical data, including theoretical analysis of representation learning dynamics and convergence properties, remains an open research challenge

Our evaluation is conducted on MIMIC-IV, a single-center, ICU-enriched database from Beth Israel Deaconess Medical Center. While this dataset provides substantial sample size and diverse clinical information, practice patterns, documentation styles, case mix, and coding practices may differ across institutions. Validation of GDP’s architecture and training strategy on multi-institutional cohorts, including those with different EHR systems, non-ICU patient populations, and varying levels of data completeness, is an essential next step. Datasets such as eICU^31^, MIMIC-III^32^, and other publicly available EHR databases represent valuable resources for such validation studies.

GDP currently uses randomly initialized embeddings for ICD diagnosis and procedure codes, which are learned jointly during pre-training. Pre-trained ICD code embeddings derived from medical ontologies, code co-occurrence patterns, or hierarchical code structures [4–5] could potentially enhance the model’s ability to capture semantic relationships between diagnoses and leverage external medical knowledge.

Importantly, both GDP variants significantly outperformed existing baselines in most metrics, indicating that our multimodal fusion strategy and pretraining regimen are fundamentally sound and beneficial. The incorporation of temporal modeling tasks (NTP, MFP) during pretraining is a key strength^33^. These tasks forced the model to learn the underlying structure of the patient timeline (e.g., that certain events follow others in time, that certain labs correlate with specific diagnoses, etc.), rather than treating the structured data as an uncorrelated bag of features. This likely contributed to the robust performance of GDP-Non-Instruct in predictions, unlike prior EHR Transformers (CEHR-BERT) that only rely on sequence position embeddings, GDP’s encoders explicitly learned to predict unseen or next events, imbuing a sense of chronological order and progression. Even for GDP-Instruct, which ultimately shined in text generation, those same pretraining tasks improved the factual grounding of its outputs. We see evidence of this in the human evaluation: GDP-Instruct had remarkably high faithfulness scores, suggesting it seldom hallucinated or omitted key facts. We attribute this to the model’s cross-attention mechanism which ties generation to the patient’s data, and to the auxiliary objectives that ensured the structured data representation is rich and descriptive of the patient. In contrast, a large generic LLM (even one tuned on medical text) might produce a fluent summary but could wander or embellish if not tightly anchored; GDP-Instruct, having been trained to generate from data, was less prone to such errors.

Our evaluation focused on binary classification tasks for diagnosis prediction. Extending GDP to multi-class disease subtyping (e.g., distinguishing heart failure with preserved vs. reduced ejection fraction), multi-label comorbidity prediction, continuous risk regression, and survival analysis would provide a more comprehensive assessment of the model’s capabilities. Such extensions are important directions for future work.

Despite these strengths, our study has several limitations that suggest avenues for future work. First, the context length of the LLM (and the practical need to limit input size to 128k) constrained how much of a patient’s history we could use. In fine-tuning, we restricted structured sequences to 50 time steps and focused on a single key clinical note (e.g., discharge summary) per admission. Patients with longer or more complex histories may have relevant information beyond these limits. This is a general challenge as EHRs can span hundreds of events and dozens of notes - future models might incorporate longer context handling (through hierarchical encoding^34^, or retrieval mechanisms^35^) to ensure no pertinent data is dropped. Second, our evaluations were on a single-center dataset (MIMIC-IV) of ICU and hospital inpatients. Relatedly, we only tackled three prediction tasks (readmission, two diagnoses) and one type of summarization. These were chosen as representative tasks, but they do not cover all possible clinical questions. The model’s utility on other targets (e.g., length of stay regression, adverse event prediction, procedure recommendation, etc.) should be explored. Third, model size and efficiency are considerations. Our LLM backbone was 3.2 billion parameters, relatively small by modern standards, primarily due to computational constraints in training a multimodal model end-to-end. It is possible that larger LLMs (e.g., 8B, 13B, or beyond) would yield even better performance, especially on generative tasks, as suggested by the baseline scaling trends (the 8B model was competitive, though it lacked our EHR-specific pretraining). However, training and deploying such large models in healthcare settings is challenging. From a deployment perspective, GDP’s current architecture is computationally intensive and not yet optimized for resource-constrained or latency-sensitive clinical environments. Future work should investigate lightweight variants, including adapter-based fine-tuning, quantization, and efficient attention mechanisms, to enable practical deployment in hospital settings where millisecond-level response times may be required for bedside decision support. Future research might investigate knowledge distillation or adapter-based tuning to compress the GDP model or deploy it in parts (for instance, a lightweight encoder at the bedside that feeds into a cloud-based LLM). Additionally, the current architecture uses a full cross-attention integration of structured data at each layer of the LLM, which is computationally expensive. More efficient fusion mechanisms (such as intermittent cross-attention or gating, or using an encoder–decoder design where the decoder attends to a fixed embedding sequence) could reduce latency and memory usage, which are important for practical use.

Another limitation is that our multimodal scope was restricted to structured EHR data and text. EHRs often contain other data types like medical images (radiology scans), waveforms (ECG, EEG), and possibly genomics^36,37^. GDP’s architecture could, in theory, be extended with additional encoders for these modalities (and some recent works do explore multimodal foundation models, including imaging), but we did not incorporate or test those. The performance on external EHR datasets from different hospital systems, and GDP’s robustness to distribution shift or missing data inputs is not assessed.

Looking ahead, several future directions emerge. One is to explore the zero-shot and few-shot learning potential of GDP. As an instruction-tuned LLM fused with clinical data, GDP-Instruct might be able to handle new tasks via prompting, without additional fine-tuning, as shown in many existing studies^38,39^. For example, given a patient record, could we prompt it with an instruction like, “List this patient’s active problems and suggested management plan,” and get a sensible result? This would move toward more flexible AI assistants that don’t require a new supervised model for every task. Preliminary chat experiments are encouraging in this regard, but systematic evaluation on benchmark clinical Q&A datasets or few-shot prediction tasks (like those in the recent EHRSHOT challenge) would be informative. Another direction is to incorporate reinforcement learning from human feedback (RLHF) to further refine the model’s generative behavior^40^. Our human evaluation gave us insight into what clinicians prefer; using that as a signal to fine-tune GDP-Instruct (similar to how ChatGPT is refined) could improve its utility and safety in an interactive setting. Additionally, exploring modular or lightweight encoder updates is worthwhile. For instance, if a hospital has an existing GDP model, how can it be efficiently adapted to local data? Techniques like Low-Rank Adaptation (LoRA) or adapter modules inserted into the GDP architecture might allow fine-tuning on a new dataset without retraining the whole model^41^. Finally, it will be important to test GDP in a prospective simulation or clinical trial context: e.g., have the model generate summaries or risk predictions on live hospital data and measure outcomes such as physician satisfaction, documentation time saved, or diagnostic accuracy when assisted by the model. These real-world evaluations will ultimately determine if models like GDP can transition from research proof-of-concept to trustworthy tools in everyday healthcare.

## Methods

### Data source and cohort

**Fig. 4:**
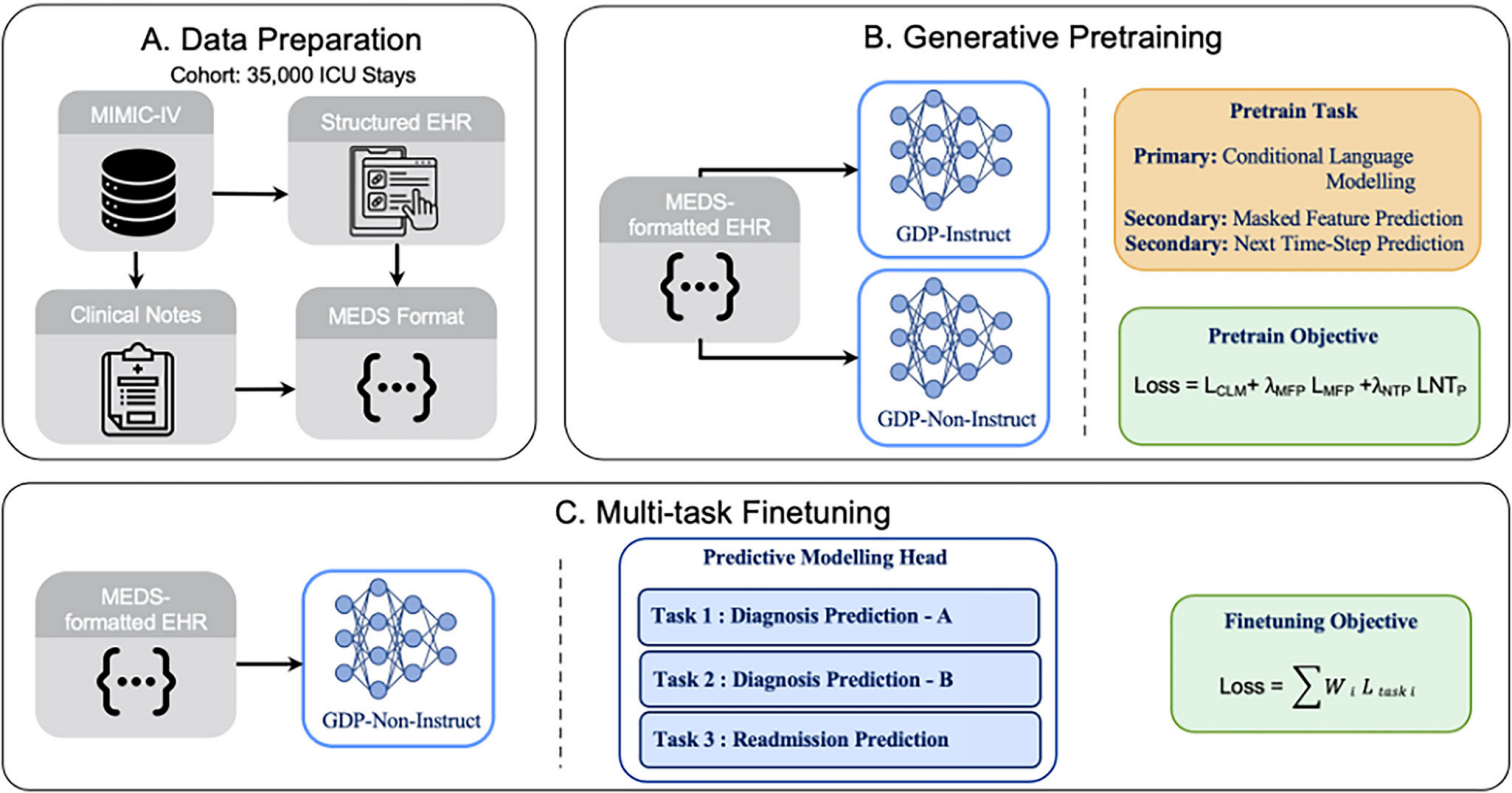
Overview of data processing and experimental pipeline. **A** shows data preparation steps. **B** shows generative pretraining stage. **C** shows multi-task finetuning stage.

Figure 4A depicts an overview of the data preparation step. We developed and evaluated our model using the Medical Information Mart for Intensive Care IV (MIMIC-IV) dataset that is available through PhysioNet with credentialed access. MIMIC-IV comprises de-identified health records for patients admitted to the ICU at a large academic medical center. For this study, we used MIMIC-IV data comprising 34,627 hospital admissions from 26,540 unique patients between 2008 and 2019^42^. Each patient encounter was represented as a combination of structured events and text. We prepared the data in the Medical Event Data Standard (MEDS) format (inspired by prior MIMIC-Extract pipelines), where each line is a JSON record containing all relevant information for one patient admission^43^. In structured data, we included: demographics (age, sex), diagnoses and procedures (ICD-9, CPT-4), laboratory test results (LOINC), medication administrations (RxNorm), and measurements such as vital signs, input/output and ventilator settings. All of these data are time-stamped. Each event entry consisted of a timestamp and a feature (or code) with an associated value (for labs/vitals) or category. Each unit of analysis corresponded to a single hospital admission. We split the dataset into training (70%), validation (15%), and holdout test (15%) sets at the patient level, ensuring no patient’s records appeared in more than one set. Exact split sizes are reported in Table 5. These events were sorted chronologically to form the patient’s timeline. In text data, we included the content of clinical notes— specifically, we leveraged discharge summaries as they provide a synopsis of the entire hospital stay.

**Table 5:** Dataset statistics and task-specific cohort sizes

Category	Subcategory	Count
Pre-training Cohort	Unique patients	26,540
	Total admissions	34,627
	Admissions with discharge summaries	31,164
Heart Failure Prediction	Total eligible admissions (after prevalent-case exclusion)	18,247
	Training set	12,773 (positive: 1,405; 11.0%)
	Validation set	2737 (positive: 301; 11.0%)
	Test set	2737 (positive: 301; 11.0%)
Type 2 Diabetes Prediction	Total eligible admissions (after prevalent-case exclusion)	16,853
	Training set	11,797 (positive: 1,652; 14.0%)
	Validation set	2528 (positive: 354; 14.0%)
	Test set	2528 (positive: 354; 14.0%)
30-day Readmission Prediction	Total eligible admissions	34,627
	Training set	24,239 (positive: 3,636; 15.0%)
	Validation set	5194 (positive: 779; 15.0%)
	Test set	5194 (positive: 779; 15.0%)

### Ethics statement

Use of the MIMIC-IV database was approved by the institutional review boards of the Massachusetts Institute of Technology and Beth Israel Deaconess Medical Center, with a waiver of informed consent because the dataset is de-identified. All methods were carried out in accordance with relevant guidelines and regulations.

### Cohort definition and handling of prevalent cases

For HF and T2DM prediction tasks, we aimed to predict incident diagnosis rather than detect prevalent conditions. We therefore applied the following exclusion criteria:

- Heart Failure cohort: We excluded all admissions where any ICD-9 code beginning with 428 or any ICD-10 code beginning with 150 appeared in the diagnosis records prior to or during the index admission. Only admissions where the first documented HF diagnosis occurred after the prediction window were included as positive cases. Admissions with no subsequent HF diagnosis were included as negative cases.
- Type 2 Diabetes cohort: We excluded admissions where ICD-9 code 250.x0 or 250.x2 (type 2 diabetes) or ICD-10 code E11 appeared in diagnosis records prior to or during the index admission. Positive cases were defined as admissions where T2DM was first documented after the prediction window.
- 30-day readmission: All admissions were eligible. Readmission status was determined by whether the patient had any subsequent admission within 30 days of discharge from the index admission.

This approach ensures that our predictive performance reflects the model’s ability to identify patients at risk of developing new diagnoses, rather than detecting already-documented conditions.

### ICD coding systems

MIMIC-IV contains diagnosis codes in both ICD-9-CM and ICD-10-CM formats, with ICD-9 codes predominating for earlier admissions (prior to October 2015) and ICD-10 codes for later admissions. For heart failure, we included ICD-9 codes beginning with 428 (428.0–428.9) and ICD-10 codes beginning with I50 (I50.0–I50.9). For type 2 diabetes, we included ICD-9 codes 250.x0 and 250.x2 (where x represents the fourth digit 0–9 indicating absence or presence of complications, and the fifth digit 0 or 2 indicates type 2) and ICD-10 code E11 (E11.0-E11.9). No additional mapping or harmonization was required as we used code prefix matching that captures equivalent diagnoses across both coding systems.

Multiple ICD code groupings have been used to define heart failure in EHR research^44,45^. We adopted a commonly used definition based on ICD-9 codes 428.x and ICD-10 codes I50.x, consistent with prior MIMIC-IV studies. Alternative definitions incorporating additional specificity (e.g., I11.0, I13.0, I13.2 for hypertensive heart failure) may capture different patient subpopulations and represent valuable directions for sensitivity analyses.

### Discriminative and generative tasks

For training the generative model, we parsed discharge summaries to extract the Brief Hospital Course (BHC) section (a narrative paragraph summarizing the hospitalization). The BHC section was used as the target text for generation. For multi-task prediction, we defined three binary outcome labels based on the EHR data: 30-day readmission (unplanned readmission to hospital within 30 days of discharge), HF diagnosis (whether the patient had a diagnosis code for HF, ICD-9 428.x, during admission), and T2DM (ICD-9 250.x for type 2 diabetes). A positive label for HF or T2DM indicates that condition was present (per coding or problem list) during the hospitalization. For prediction tasks, we used data available at the time of admission (early hospital events) to predict HF, DM, or 30-day readmission risk. For narrative generation, the model used the entire admission timeline to generate the BHC at discharge. The remaining tasks had a mix of positive and negative cases (prevalence: HF ~ 11%, T2DM ~ 14%, readmission ~15%). We split the dataset into training, validation, and holdout test sets at the patient level (so no patient’s records appear in more than one set). The test set was about 15% of the data (thousands of admissions for each task). All structured features were normalized or encoded before input: continuous values (lab results, etc.) were z-scored or bucketed, and categorical codes were mapped to integer indices. Table 6 lists the data types and encoding used.

**Table 6:** Structured data types and encoding approaches

Data type	Encoding/handling
Age, sex (demographics)	Directly encoded (age normalized, sex as binary categorical)
Diagnoses (ICD-9/10)	Mapped to integer indices; embedded
Procedures	Mapped to integer indices; embedded
Lab results (numeric)	Z-scored or bucketed into ranges
Lab results (categorical, e.g., positive/negative)	One-hot encoded/mapped to integer
Medications – oral/tablets (scheduled)	Encoded as discrete events with time gaps
Medications – IV/continuous	Represented as time-stamped events with duration
Vital signs (BP, HR, RR, etc.)	Continuous values; forward-filled within windows
Input/output measures	Continuous values; normalized
Ventilator settings and other device parameters	Continuous/categorical as appropriate

### Data preprocessing

All data were processed through a standardized pipeline to prepare model inputs. Structured event sequences: Each patient’s timeline of events was transformed into a fixed-length sequence of vectors for modeling. We first defined a vocabulary of the most frequent coded events (diagnoses, procedures, medications, lab types, etc.) and allocated an embedding vector for each code. For each timestamp in a patient’s record (e.g., each hour or each day with recorded data), we aggregated the events occurring at that time into a single vector. Specifically, if multiple codes occurred at the same time, their embeddings were averaged; associated numeric values (like lab results) were appended as scaled features. We also appended time features such as the time since admission for each event. If a sequence had more events than the maximum length (we set max length = 100 for fine-tuning, based on distribution of stay lengths), we truncated it to the most recent 50 events; if shorter, we padded it with dummy vectors. This resulted in a [*T*×*d*] matrix per patient (with *T* = 100, *d* = embedding dimension, e.g., 128), representing the structured data timeline. We applied minor data cleaning, for example filtering out rarely-used codes and forward-filling missing values for vitals within short windows. Text data: The discharge summary (or specifically the BHC section) was tokenized using a subword tokenizer (the same tokenizer as the LLM backbone). We limited the length to a set number of tokens (e.g., 256 tokens) to fit the model’s context window. In generative pretraining, the structured data sequence served as context and the full BHC text was the target to generate. For fine-tuning tasks, we also processed notes: for each admission, we took the text of the discharge summary (minus the BHC) as additional input to the model’s text encoder (described below). All text was lowercased and de-identified (MIMIC is already de-identified; we further removed any surrogate identifiers). No other text normalization was done; the clinical language was kept in its original form to preserve meaning.

### Model architecture

**Fig. 5:**
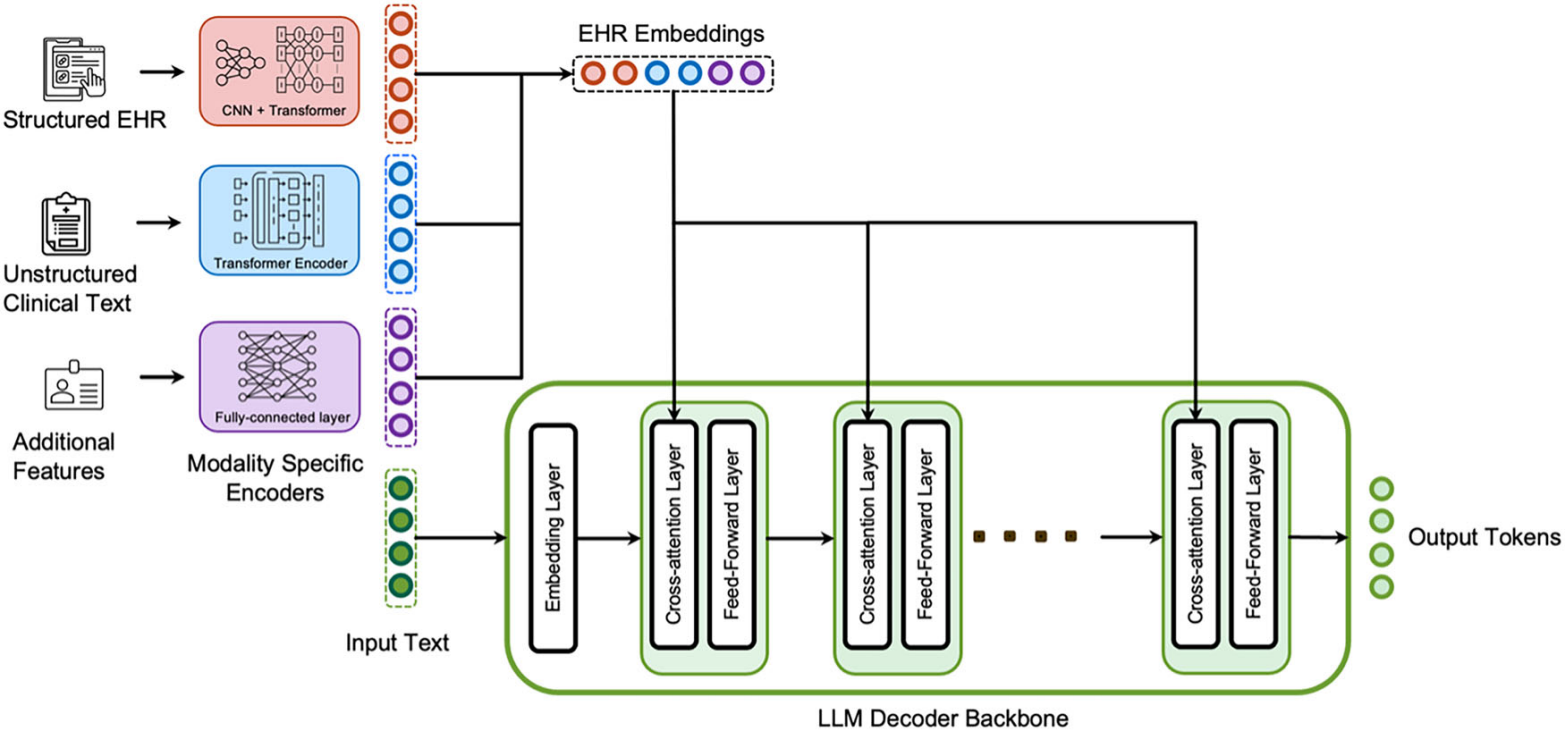
Detailed GDP model components. The GDP model consists of three core components: (1) a structured data encoder for processing tabular and coded clinical data, (2) an unstructured text encoder for capturing information from clinical narratives, and (3) an LLM backbone that performs multimodal fusion of structured and unstructured representations. On top of this architecture, the model incorporates task-specific output heads that support both generative and classification tasks.

The GDP model is a multimodal encoder–decoder architecture built around a Transformer-based LLM. Figure 5 depicts its components. There are three main subsystems: (1) the Structured Data Encoder, (2) the Unstructured Text Encoder, and (3) the LLM backbone with multimodal fusion. Additionally, the model has task-specific output heads for generation and classification.

### Structured data encoder

This module processes the sequence of structured EHR event vectors. It begins with several 1-D convolutional layers that scan over the time dimension of the event sequence. These CNN layers capture local temporal patterns and smooth out high-frequency noise in the sequences (for example, detecting a short trend in vital signs)^46^. Next, the encoder includes Transformer encoder layers (self-attention blocks) that operate over the sequence length *T*. The Transformer layers enable learning of long-range dependencies across the entire timeline, for instance, attention can connect an event early in the hospitalization with one much later, if relevant. We used positional encodings to preserve the order of events in these attention layers. The structured encoder produces a sequence of hidden vectors of length *T*. We projected the final outputs to a hidden dimension of 256 (so each time step is represented by a 256-d embedding). If present, a separate small Feature Encoder processes any auxiliary features (like static demographics or time-gap features) via a simple feed-forward network, and outputs a 256-d embedding that can be added to or concatenated with the main sequence representation. The design choice of a hybrid CNN+Transformer for structured data balances efficiency and expressiveness: CNN layers rapidly reduce noise and extract short-term trends, while Transformers capture global temporal context. This is advantageous over relying solely on recurrent networks, which can be slower and may struggle with long sequences.

Each patient timeline consists of 100 hourly embeddings with 128 dimensions per embedding. The CNN component consists of three sequential convolutions, each with a kernel size of 3, a stride of 1, and a padding of 1. The first convolutional layer maintains 128 channels and is followed by batch normalization and ReLU activation. The second layer expands to 256 channels, also with batch normalization and ReLU activation. The third layer maintains 256 channels but employs layer normalization followed by GELU activation. The CNN produces a sequence representation of dimensions $100\times 256\,$.

The Transformer component then processes the CNN output. Learned positional embeddings of matching dimensions are added to the sequence representation before passing it through four Transformer encoder layers. Each Transformer layer utilizes a hidden dimension of 256, 8 attention heads, a feed-forward network with an inner dimension of 512, and a dropout rate of 0.1, with pre-normalization architecture. The final output maintains the dimensions of $100\times 256\,$. A 256-dimensional demographic embedding is added to each time step before fusion with other modalities, ensuring incorporation of patient-specific static features.

Each patient timeline consists of 100 hourly embeddings (128 D). A 1D-CNN front end applies three convolutions (kernel = 3, stride = 1, padding = 1):

- Conv1: 128 → 128 channels, BatchNorm → ReLU
- Conv2: 128 → 256 channels, BatchNorm → ReLU
- Conv3: 256 → 256 channels, LayerNorm → GELU

This produces a sequence of shape [100 × 256]. Learned positional embeddings (100 × 256) are added, and the result is passed through four Transformer encoder layers (hidden = 256, 8 heads, FFN = 512, dropout = 0.1, pre-norm). The final output is [100 × 256]. A 256-D demographic embedding is added to each time step before fusion.

### Unstructured text encoder

For clinical text inputs (e.g., discharge notes), we employed a pretrained clinical BERT encoder. Specifically, we used the BioClinicalBERT model (a BERT-base model further pre-trained on biomedical and clinical text) as our text encoder^4^. This encoder takes tokenized text (up to ~512 tokens) and produces a contextual embedding. We take the [CLS] token embedding (a 768-d vector) from the BERT encoder as a summary of the note’s content. A linear projection then maps this to the 256-d hidden space. During model training, this text encoder can be fine-tuned or kept frozen depending on the stage—in generative pretraining, we did update it when generating text conditioned on text+structured data; in fine-tuning, we typically froze BERT initially and then allowed fine-tuning in later epochs. By leveraging a pretrained language model here, we provide GDP with a strong understanding of medical language out of the box, so it can comprehend clinical narrative context and terminology.

### LLM backbone and fusion

At the core of GDP is a transformer decoder architecture based on Meta’s LLaMA model. We used a custom 3.2 billion-parameter LLaMA-like model as the backbone. For GDP-Non-Instruct, this backbone was initialized from a standard pre-trained LLaMA model (trained on general text but not instruction-tuned). For GDP-Instruct, we initialized from an instruction-tuned variant of LLaMA of the same size— essentially a model that had gone through an additional round of fine-tuning on instructional datasets (similar to how Alpaca or LLaMA-2 Chat models are derived). The backbone has the typical structure of a decoder-only transformer (layers of self-attention and feed-forward blocks), augmented with cross-attention to incorporate multimodal inputs. Specifically, we modified each transformer layer to include a cross-attention sublayer that attends to the sequence of structured data encoder outputs. In practice, the structured data encoder’s final hidden states (the 50 × 256 sequence) are treated as “memory” for the LLM. At each generation step, the LLM’s cross-attention heads can attend to any of those memory vectors, thereby allowing the content of the patient’s timeline to influence the generated tokens. This mechanism is analogous to an encoder–decoder Transformer in machine translation, except here the “encoder” is the structured data module.

Additionally, to fuse information from text notes when available (during fine-tuning for prediction), we employed a simple strategy: the 256-d note embedding from the text encoder is added to each time-step embedding of the structured sequence (broadcast across all positions) before it is fed into the cross-attention. This effectively informs the model that, for example, “this patient’s sequence corresponds to someone whose note embedding is X,” allowing the LLM to modulate its attention based on both structured and unstructured context. We also experimented with concatenating the note embedding as an additional token in the structured sequence, which yielded similar results. A layer normalization is applied to the fused sequence. The cross-attention layers in the LLM use multi-head attention to query this fused sequence from each decoder token position. In summary, during the generative mode, the LLM backbone takes as input the structured (and any text) context and generates a sequence of text tokens (the BHC) autoregressively, with cross-attention providing grounding. During the predictive mode, the LLM can be thought of as processing the structured (and text) data into an integrated latent representation (although in fine-tuning we do not actually *generate* text, we instead use the LLM’s latent state for classification as described next).

Each decoder consists of 24 layers incorporating masked self-attention, cross-modal attention, and feed-forward components. The masked self-attention mechanism employs a hidden dimension of 256, 16 attention heads, causal masking, and a dropout rate of 0.1. The cross-attention mechanism processes queries from the self-attention 256-dimensional output against keys and values derived from the fused embeddings ($100\times 256\,$ dimensions), utilizing 8 attention heads and a dropout rate of 0.1. The feed-forward network expands the representation from 256 to 1024 dimensions before projecting back to 256, employing GELU activation and a dropout rate of 0.1.

LayerNorm is applied before each sublayer, with residual connections employed throughout the network. The vocabulary remains the original LLaMA 32,000-token BPE. During autoregressive generation, the decoder attends to the fused EHR embeddings at every generation step. For classification tasks, the hidden state corresponding to the beginning-of-sequence token is utilized.

We use two LLaMA-3.2B variants:

- GDP-Non-Instruct (raw 3.2 B checkpoint)
- GDP-Instruct (instruction-tuned variant)

Each of the 24 decoder layers comprises:

1. Masked self-attention (hidden = 256, 16 heads, causal mask, dropout = 0.1)
2. Cross-attention (queries = 256-D from self-attention; keys/values = [100 × 256] fused embeddings; 8 heads, dropout = 0.1)
3. Feed-forward (256 → 1 024 → 256, GELU, dropout = 0.1)

Preceding each sublayer is LayerNorm, and residual connections are employed. Vocabulary remains LLaMA’s 32,000-token BPE. During autoregressive generation, the decoder attends to fused EHR embeddings at every step. For classification, the hidden state at the <BOS> token is used.

For multimodal fusion, we adopt a two-stage strategy. Within-timestamp fusion: Multiple events of the same modality occurring at the same timestamp (e.g., multiple laboratory tests) are encoded individually and then averaged to produce a single modality-specific representation per timestamp. This averaging operation provides a simple, stable aggregation that is invariant to event ordering within a timestamp. Cross-modality fusion: Modality-specific representations at each timestamp are concatenated and projected through a linear layer to form a unified EHR representation. This representation is then passed to cross-attention layers where it interacts with text encoder outputs.

We explored alternative fusion strategies during development, including: (1) learned attention pooling within timestamps, which showed marginal improvements (~0.5% AUC) at the cost of increased training instability and memory usage, and (2) modality-specific cross-attention before temporal fusion, which did not yield consistent performance gains across tasks. We selected the current architecture for its balance of expressiveness, computational efficiency, and training stability. More sophisticated fusion mechanisms, such as gated fusion, multi-head cross-modal attention, or graph-based fusion over event relationships, represent promising directions for future architectural refinement

### Multi-task output heads

For the predictive tasks, we added a lightweight classification head on top of the LLM backbone. After feeding the patient’s data through the structured/text encoders and into the LLM (with no text prompt given to generate), we obtain the LLM’s final hidden state for the sequence. We take the hidden state corresponding to a special beginning-of-sequence token (analogous to a [CLS] token) as an aggregate representation of the patient. This 256-d vector is then fed into a set of parallel linear classifier layers -one for each prediction task. Each classifier outputs a logit (for binary tasks), which is passed through a sigmoid to get a predicted probability. In our case, we had three classifiers (readmission, HF, T2DM). The multi-task head thus shares the backbone representation but produces task-specific outputs. This design allows the model to leverage commonalities among tasks (e.g., features predictive of T2DM might overlap with those predictive of readmission) while still giving flexibility for each task’s decision boundary. During fine-tuning, the model computes all task losses and sums them (with weighting) to update the shared layers. For the generative task, the output head is simply the LLM’s native causal language modeling (LM) head—a linear layer tying into the LLM’s vocabulary, used to predict the next token at each step of text generation.

Overall, GDP’s architecture enables two training modes: a generative mode (structured data in, text out) with cross-attention guiding the text generation, and a classification mode (structured data in, label out) using the fused representation. The instruction-tuned vs non-tuned backbone differs only in initialization (and subsequent fine-tuning dynamics). By loading weights from a pre-trained LLaMA or LLaMA-Instruct model, we gave GDP a strong language foundation at the start, which we expected to help with textual coherence and general knowledge. The cross-attention layers ensured that this language model doesn’t operate blindly, but rather always in context of the patient data—crucial for factual accuracy in generation.

### Model training

Generative pretraining stage, as shown in Fig. 4 (B): In the first stage, we trained the GDP model to model the joint distribution of EHR data and clinical text. Each training sample consisted of a patient’s structured sequence (and note embedding, if note present) as context input, and the BHC text as the target output. The model was optimized to generate the BHC text from the context. We used a standard autoregressive language modeling loss (cross-entropy loss on each generated token) for this^47^. Specifically, we minimize the standard autoregressive cross-entropy:

1 $${L}_{{LM}}=-\mathop{\sum }\limits_{t=1}^{L}\log \,{P}_{\theta }({w}_{t}{\rm{| }}{w}_{ < t},{EH}{R}_{{fused}})$$

where *w*_*t*_ are tokens of the Brief Hospital Course and Here, *L* ≤ 256 and *EHR*_*fused*_ denotes the 100×256 cross-attention memory.

In addition, we simultaneously trained two auxiliary objectives on the structured data encoder: Masked Feature Prediction (MFP) and Next Time-step Prediction (NTP). For MFP, we randomly masked out a small fraction of the structured input vectors (or certain features within those vectors) and asked the model to reconstruct them. Specifically, we zeroed out some event embeddings and had a small decoder network predict the original values. The MFP loss was the mean squared error between the reconstructed and true feature values (or cross-entropy for masked code predictions). For NTP, for each sequence we withheld the final event vector and tasked the model to predict it given all prior events.

2 $${L}_{{MFP}}=\frac{1}{{|M|}}\mathop{\sum }\limits_{i\in M}{||}{x}_{i}-{{\hat{x}}_{i}{||}}^{2}$$

3 $${L}_{{NTP}}={||}{h}_{T}-{x}_{T+1}{{||}}^{2}$$

where,$\left|{\mathcal{M}}\right|$ is the set of masked positions in the structured sequence.${x}_{i}$ is the original event embedding, $\hat{{x}_{i}}$ its reconstruction.${h}_{T}$ is the encoder’s final hidden state for the first T events, and ${x}_{T+1}$ the true next-event embedding.

This was implemented via another feed-forward layer that takes the encoder’s final hidden state (after CNN+Transformer) and outputs a prediction of the next event embedding; the NTP loss was the MSE between this prediction and the actual next-event vector.

We weighted these losses with coefficients λ_MFP_ and λ_NTP_ in the total pretraining loss. The overall pretraining objective was:

4 $${L}_{{\mathrm{Pretrain}}}={L}_{{\mathrm{CLM}}}+{\lambda }_{{\mathrm{MFP}}}\,{L}_{{\mathrm{MFP}}}+{\lambda }_{{\mathrm{NTP}}}\,{L}_{{\mathrm{NTP}}}$$

We set $\lambda \mathrm{MFP}$ = $\lambda \mathrm{NTP}$ = 1 initially (treating all losses equally), and we annealed them slightly down later in training to focus on LM loss as it improved. We used the AdamW optimizer with a learning rate of ~2e-4 for the backbone and 1e-3 for scratch parameters, and a linear warmup of a few thousand steps. Training was done on A100 GPU(80 GB) in mixed precision (fp16), with gradient accumulation such that the effective batch size was about 8 patient samples per update (each containing one note to generate). We trained until validation perplexity stopped improving (around 5 epochs through the data). The generative pretraining took around 2 days. Both GDP-Instruct and GDP-Non-Instruct underwent this pretraining (with identical hyperparameters), resulting in two pretrained model checkpoints.

Multi-task fine-tuning stage, as shown in Fig. 4C: In the second stage, we fine-tuned each pretrained model on the supervised prediction tasks. We froze the LLM backbone weights initially to avoid catastrophic forgetting of the language modeling capability. In the first 1–2 epochs of fine-tuning, we only trained the newly initialized components: the multi-task classification head and the fusion projections. We also unfroze the structured data encoder to adapt it to the new task signals. Then, we gradually unfroze the LLM backbone layers: over the next few epochs, we incrementally allowed more transformer layers to be trainable (starting from the top layers). This layer-freezing schedule, combined with a lower learning rate for the LLM layers (we used a 5× lower LR for backbone compared to encoders and heads), helped stabilize training. We fine-tuned using the AdamW optimizer (LR ~5e-5 for head, ~1e-5 for backbone). The loss function for fine-tuning was the sum of each task’s binary cross-entropy loss. To handle class imbalance, we applied class-weighted focal loss for some tasks^48–50^. In practice, for readmission (where positives are relatively rare), using focal loss (*γ* = 2) improved recall. The total fine-tune loss was

5 $${L}_{{\mathrm{finetune}}}=\sum {W}_{i}\,{L}_{{{task}}_{i}}$$

where *w*_*k*_ are task weights (we set them to 1 for each task). We trained in a multi-task fashion: each batch contained examples with all task labels (if a label was missing for a patient, we masked that loss). Fine-tuning continued for ~10 epochs, with early stopping based on validation AUROC to prevent overfitting. The final chosen model was the epoch with highest average AUROC across tasks on the validation set. We repeated fine-tuning for both GDP-Instruct and GDP-Non-Instruct, starting from their respective pretrained weights.

For the summarization task (BHC generation), we fine-tuned GDP-Instruct and GDP-Non-Instruct separately on note generation as well (though they had already seen that task in pretraining). We found that a brief additional fine-tuning on the smaller supervised set of paired structured data–BHC (using only the LM loss) helped alignment with the reference style and improved ROUGE slightly. The baselines (Meta Llama and Med42) were also fine-tuned on this task using the same hyperparameters for a fair comparison.

### Training configuration

GDP is trained on a single NVIDIA A100 GPU (80GB) for approximately 48 h for generative pretraining, completing approximately 5 epochs over the pre-training cohort with a batch size of 8 (effective, via gradient accumulation) and a maximum sequence length of 512 tokens for text and 100 time steps for structured EHR. The computational cost is driven primarily by: (1) full cross-attention between EHR encoder outputs and text encoder outputs at each of 24 decoder layers, and (2) autoregressive decoding during discharge summary generation pre-training.

GDP is trained using a two-staged approach: generative pretraining followed by supervised fine-tuning. For all training stages, we use the AdamW optimizer with ${\beta }_{1}=0.9$, ${\beta }_{2}=0.95$, $\epsilon ={10}^{-8}$ and weight decay of 0.01. The learning rate follows a cosine schedule with linear warmup.

During generative pretraining, we use an effective batch size of 8 through gradient accumulation of four micro-batches of size 2. The backbone learning rate is set to $2\times {10}^{-4}$ while encoders and prediction heads use a higher rate of $1\times {10}^{-3}$. The learning rate warms up linearly over the first 1000 steps and then decays following a cosine schedule to zero over 50,000 steps (approximately 5 epochs). This stage is conducted using mixed precision on A100 GPUs with early stopping if validation perplexity on BHC fails to improve for two consecutive epochs.

For multi-task fine-tuning, we simultaneously optimize for three clinical prediction tasks: Heart Failure (HF), Type 2 Diabetes (T2DM), and 30-day Readmission. The beginning-of-sequence (BOS) hidden state with 256 dimensions feeds three parallel classification heads, each consisting of a linear layer followed by sigmoid activation. We use a batch size of 16 with learning rates of $5\times {10}^{-5}$ for fusion components and classification heads, and $1\times {10}^{-5}$ for the backbone. The fine-tuning loss is defined as:

$$[ \mathcal{L}{\mathrm{fine}} = w{\mathrm{HF}} , {\mathrm{BCE}}(p_{\mathrm{HF}}, y_{\mathrm{HF}}) w_{\mathrm{T2DM}} , {\mathrm{BCE}}(p_{\mathrm{T2DM}}, y_{\mathrm{T2DM}}) w_{\mathrm{Readm}} , {\mathrm{Focal}}(p_{\mathrm{Readm}}, y_{\mathrm{Readm}})]$$

where BCE refers to Binary Cross-Entropy loss, ${w}_{{HF}}=0.90$, ${w}_{{T2DM}}=0.86$, and ${w}_{{Readm}}=0.85$ are the balancing hyperparameters. Focal Loss hyperparameters are set to $\gamma =2\,$ and $\alpha =0.25\,$.

To prevent overfitting during fine-tuning, we implement a progressive unfreezing strategy. During the first two epochs, the decoder remains frozen. For epochs three through five, we unfreeze the top six layers. Finally, all parameters are trainable for the remaining epochs. We use early stopping based on mean validation AUROC with a patience of three epochs and a maximum of ten epochs.

### Generative pretraining

Optimizer: AdamW (backbone LR = 2 × 10⁻⁴; encoders + heads LR = 1 × 10⁻³; weight decay = 0.01; *β*₁ = 0.9, *β*₂ = 0.999, *ε* = 10⁻⁸). Warmup: linear over 1000 steps; cosine decay to zero over 50,000 steps (~5 epochs). Batch size: effective = 8 (gradient accumulation of four micro-batches of 2). Mixed precision (FP16) on A100 GPUs. Early stopping if validation perplexity on BHC fails to improve for two consecutive epochs.

### Multi-task fine-tuning

Tasks: HF, T2DM, and 30-day Readmission. The <BOS> hidden state (256 D) feeds three parallel linear → sigmoid heads. Loss:

6 $${L}_{{fine}}={w}_{{HF}}\,{BCE}\left({p}_{{HF}},{y}_{{HF}}\right)+{w}_{T2{DM}}\,{BCE}({p}_{T2{DM}}),{y}_{T2{DM}})+{w}_{{Readm}}\,{Focal}({p}_{{Readm},}\,{y}_{{Readm}})$$

Optimizer: AdamW (fusion + heads LR = 5 × 10⁻⁵; backbone LR = 1 × 10⁻⁵; weight decay = 0.01). Freezing schedule: epochs 1–2 freeze decoder, epochs 3–5 unfreeze top 6 layers, epochs 6–10 unfreeze all. Batch size = 16. Early stopping on mean validation AUROC (patience = 3, max epochs = 10).

### Evaluation metrics and analysis

We evaluated classification performance on the test set using several metrics. Discrimination was measured by the area under the ROC curve (AUROC) and area under the precision–recall curve (AUPRC), computed for each task. These threshold-independent metrics summarize the model’s ability to rank-order predictions. We calculated 95% confidence intervals for AUROC/AUPRC via bootstrapping 1000 samples on test set. Threshold-dependent metrics were computed at the conventional 0.5 probability cutoff (since we had balanced validation sets, this was reasonable). We report F1score, precision, recall, and accuracy for each model on each task (Table 1). F1 is the harmonic mean of precision and recall, reflecting a balance between sensitivity and specificity.

7 $${Precision}=\frac{{TP}}{{TP}+{FP}}$$

8 $${Recall}=\frac{{TP}}{{TP}+{FN}}$$

9 $${F}_{1}=2\times \frac{{\mathrm{Precision}}\times {\mathrm{Recall}}}{{\mathrm{Precision}}+{\mathrm{Recall}}}$$

10 $${Accuracy}=\frac{{TP}+{TN}}{{TP}+{TN}+{FP}+{FN}}$$

11 $${AUROC}={\int }_{0}^{1}{\mathrm{TPR}}(\left({{\mathrm{FPR}}}^{-1}(t)\right){dt}$$

12 $${AUPRC}={\int }_{0}^{1}{\mathrm{Precision}}\left({{\mathrm{Recall}}}^{-1}(r)\right){dr}$$

Where TP/TN/FP/FN denote true positives, true negatives, false positives, and false negatives, respectively. TPR = TP/(TP + FN) and FPR = FP/(FP + TN).The integrals for AUROC and AUPRC are typically estimated via trapezoidal approximation over the ROC and precision–recall curves.

For generative performance, we used standard natural language generation (NLG) metrics on the test set of discharge summary. We computed ROUGE-1, ROUGE-2, and ROUGE-L F1-scores (covering unigram overlap, bigram overlap, and longest common subsequence overlap) between the generated summaries and reference summaries. We report the F-measure variant of ROUGE, which balances precision and recall of overlap. We also calculated BLEU scores (up to BLEU-4, cumulative), which measure n-gram precision—an indicator of how exactly the model reproduced the reference phrasing. However, since strict n-gram matches can be overly harsh for this task (there can be many ways to write the same clinical fact), we included BERTScore, which uses a pre-trained language model to assess semantic similarity between the generated and reference text. BERTScore outputs a similarity score in [0,1]. All these metrics were computed using standard libraries (rouge-score, nltk for BLEU, and the BERTScore package with the recommended biomedical BERT model). We considered the reference summaries written by clinicians as the gold standard, and we averaged the scores across all test samples for each model (Table 2). To test for significance in metric differences, we used paired bootstrap resampling (e.g., for ROUGE-L differences between GDP-Instruct and baseline LLM).

13 $${ROUGE}\_N\_F1=2\times \frac{{Overlap}\_{Precision}\_N\times {Overlap}\_{Recall}\_N}{{Overlap}\_{Precision}\_N+{Overlap}{\rm{\_}}{Recall}\_N}$$

14 $${BLEU}={BP}\times \exp !\left(\mathop{\sum }\limits_{n=1}^{4}{w}_{n}\,\mathrm{ln}\,{p}_{n}\right)$$

15 $${BERTScore}\_F1=2\times \frac{{Precision}\_{BERT}\times {Recall}\_{BERT}}{{Precision}\_{BERT}+{Recall}\_{BERT}}$$

Where,

16 $${Overlap}{\rm{\_}}{{Precision}}_{N}=\frac{\sum {matched}\,N{\textstyle \mathrm{-}}{grams}}{\sum {generated}\,N{\textstyle \mathrm{-}}{grams}}$$

17 $${Overlap}{\rm{\_}}{{Recall}}_{N}=\frac{\sum {matched}\,N-{grams}}{\sum {reference}\,N-{grams}}$$

18 $${p}_{n}={Overlap}{\rm{\_}}{{Precision}}_{n}$$

19 $${{Precision}}_{{BERT}}=\frac{1}{L}\mathop{\sum }\limits_{i=1}^{L}\mathop{\max }\limits_{j}{sim}({t}_{i},{s}_{j})\,$$

20 $${{Recall}}_{{BERT}}=\frac{1}{M}\mathop{\sum }\limits_{j=1}^{M}\mathop{\max }\limits_{i}{sim}({s}_{j},{t}_{i})$$

$${BP}=\{1,{if\; c} > r$$

$$\exp (1\mbox{--}r/c),{if\; c}\le r\}$$

For human evaluation, as described in “Results”, two experts rated a set of 200 generated summaries from each model. The summaries were randomly selected such that each evaluator saw each model’s output for the *same* patient (allowing within-patient comparison across models, without knowing which was which). The rating criteria (faithfulness, completeness, conciseness, readability, and clinical utility) were each scored 1–5. We also computed Cohen’s *κ* to measure inter-rater reliability for each criterion; all *κ* were in the 0.6–0.75 range, indicating substantial agreement. No serious disagreements (difference >2 on the Likert scale) occurred on the sampled evaluations.

Finally, for the interactive chat evaluation, we did not perform a formal study but rather anecdotal trials. We ensured that the chat interface had access to the model’s weights and that the patient data was loaded in a consistent way for both GDP-Instruct and GDP-Non-Instruct. We logged example Q&A sessions for qualitative analysis. These examples were used to subjectively assess the coherence and accuracy of each model’s responses when faced with questions about the patient’s data. While not a quantitative test, it provided insights reported in the Results to illustrate the models’ capabilities in a conversational context.

All experiments were conducted in Python using PyTorch. Model training and evaluation code will be released to allow reproduction of these results.

## Supplementary information

Supplementary information

## Data Availability

The MIMIC-IV dataset used in this study is available through PhysioNet with credentialed access. Processed data in the MEDS JSONL format and task labels can be made available from the corresponding authors upon reasonable request (and in compliance with data use agreements). The discharge summary texts are part of MIMIC-IV and subject to the same access requirements.

## Code availability

Upon publication, we will release the complete codebase—including data preprocessing pipeline, model architecture implementation, pre-training procedure, fine-tuning protocols, and evaluation scripts via GitHub under an open-source license. Released code will include detailed documentation, configuration files, and example notebooks to enable full reproducibility. The MIMIC-IV dataset is available through PhysioNet (https://physionet.org/content/mimiciv/) with credentialed access, instructions for obtaining data access will be provided in the code repository.
