# Supplementary material for: A multimodal generative model for structured and unstructured electronic health records
Source: Npj Health Syst. 2026 Jun 15;3:46. doi: 10.1038/s44401-026-00095-y (PMC13269122; doi:10.1038/s44401-026-00095-y)
Supplement: Supplementary file 1 — Supplementary information [file 44401_2026_95_MOESM1_ESM.docx]

# **Supplementary Material to**

A Multimodal Generative Model for Structured and Unstructured Electronic Health Records

**Sonish Sivarajkumar^1^, Hang Zhang^1^, Yuelyu Ji^1^; Maneesh Bilalpur^1^; Xizhi Wu^2^; Chenyu Li^3,^; Min Gu Kwak^2^; Shyam Visweswaran ^1,3,4^, Yanshan Wang^1,2,3,4,5^** ^†^

*^1^Intelligent Systems Program, University of Pittsburgh, Pittsburgh, PA, USA*

*^2^Department of Health Information Management, University of Pittsburgh, Pittsburgh, PA, USA*

*^3^Department of Biomedical Informatics, University of Pittsburgh, Pittsburgh, PA, USA*

*^4^Clinical and Translational Science Institute, University of Pittsburgh, Pittsburgh, PA ^5^Hillman Cancer Center, University of Pittsburgh Medical Center, Pittsburgh, PA*

**† corresponding author: Yanshan Wang, yanshan.wang@pitt.edu**

## Key Hyperparameters

Below is a concise table summarizing the principal hyperparameters used in both pretraining and fine-tuning. For full details (including less critical settings such as tokenizer specifics and data split random seeds), refer to the code repository and configuration files.

**Table S1**. Key hyperparameters

| **Component** | **Hyperparameter** | **Value / Setting** |
| --- | --- | --- |
| Structured Encoder (CNN → Trans.) | CNN Layer 1 filters (kernel = 3) | 128 channels → ReLU |
|  | CNN Layer 2 filters (kernel = 3) | 256 channels → ReLU |
|  | CNN Layer 3 filters (kernel = 3) | 256 channels → GELU |
|  | Transformer layers | 4 |
|  | Transformer hidden dimension | 256 |
|  | Transformer heads | 8 |
|  | Transformer FFN inner dimension | 512 |
|  | Transformer dropout | 0.1 |
| Text Encoder (BioClinicalBERT) | Pretrained checkpoint | bioclinicalbert-base-cased |
|  | Projection to fusion dimension | 768 → 256 |
| LLM Decoder Backbone | Model variant | LLaMA-3.2B (Non-Instruct / Instruct) |
|  | Number of decoder layers | 24 |
|  | Decoder hidden dimension | 256 |
|  | Decoder attention heads | 16 |
|  | Decoder feed-forward dimension | 1,024 |
|  | Decoder dropout | 0.1 |
| Auxiliary Objectives | Masking ratio (MFP) | 15 % |
|  | λ_MFP | 1.0 (initial), 0.5 (after epoch 3) |
|  | λ_NTP | 1.0 (initial), 0.5 (after epoch 3) |
| Pretraining Optimizer | Algorithm | AdamW |
|  | LR (backbone) | 2×10⁻⁴ |
|  | LR (encoders + new heads) | 1×10⁻³ |
|  | Weight decay | 0.01 |
|  | Warmup steps | 1,000 |
|  | Total steps | ≈ 50,000 (∼ 5 epochs) |
|  | Effective batch size | 8 (admissions × 4 micro-batches) |
|  | Precision | FP16 (mixed-precision) |
| Fine-Tuning Optimizer | Algorithm | AdamW |
|  | LR (fusion + classifier heads) | 5×10⁻⁵ |
|  | LR (backbone) | 1×10⁻⁵ |
|  | Weight decay | 0.01 |
|  | Freezing schedule | Epochs 1–2: freeze decoder; Epochs 3–5: unfreeze top 6; Epochs 6–10: all trainable |
|  | Batch size | 16 (admissions) |
|  | Early stopping (patience) | 3 epochs (mean validation AUROC) |
| Multi-Task Loss Weights | w_HF | 0.90 (positive rate ≈ 11 %) |
|  | w_T2DM | 0.86 (positive rate ≈ 14 %) |
|  | w_Readm | 0.85 (positive rate ≈ 15 %) |
|  | Readmission loss | Focal (γ = 2, α = 0.25) |

## Ablation analysis: impact of instruction tuning and auxiliary objectives

The divergent results between GDP-Instruct(GDP with LLaMA-instruct backbone) and GDP-Non-Instruct(GDP with LLaMA-non-instruct backbone) highlight an interesting trade-off. The standard LLM backbone (Non-Instruct) proved slightly superior for purely predictive tasks, whereas the instruction-tuned backbone was markedly superior for text generation and summary tasks. This suggests that the initial training paradigm of the LLM influences what it excels at: instruction tuning endows the model with strengths in following prompts and generating fluent, contextually appropriate text (critical for narrative tasks), but the process might marginally dilute the predictive signal for classification tasks. (For clarity: fine-tuning refers to updating a pre-trained model’s parameters on a specific task or domain; instruction tuning is a type of fine-tuning where the model learns to follow human instructions across diverse commands; and prompt engineering means crafting effective inputs for an already-trained model without changing its parameters). In our experiments, GDP-Non-Instruct consistently achieved higher AUROC/AUPRC on structured tasks (Table 1), indicating a better discrimination capacity after fine-tuning, while GDP-Instruct consistently produced more accurate and readable free-text outputs (Tables 2 and 3). In practice, both variants still performed strongly on all tasks, but the choice of LLM backbone could be optimized based on the target application (analytic vs. generative). Future work may explore hybrid approaches to get the best of both – for example, further instruction-tuning the model after fine-tuning on predictive tasks, or using lightweight adapters to re-balance these capacities.

We also investigated the contribution of GDP’s auxiliary pretraining losses – the Masked Feature Prediction (MFP), inspired by Masked Language Modeling ^49^, and Next Time-step Prediction (NTP), inspired by Next Token Prediction ^50^, tasks – to its performance(Table S3). These losses were applied during generative pretraining to encourage the structured data encoder to learn richer representations. Qualitatively, we found they were vital for learning temporal relationships. Without NTP, for instance, the model’s ability to anticipate disease progression events was weakened, leading to drops in predictive accuracy. An ablation experiment where we removed the NTP loss during pretraining resulted in a noticeable decrease in AUROC (e.g., ~3–5 point drop on HF and T2DM prediction) and a flatter precision–recall curve, indicating the model was less adept at identifying patients with those conditions. MFP had a more modest but still positive effect: it helped the model capture fine-grained details in the EHR vectors, which translated to slight improvements in recall for the diagnosis tasks (by ensuring the encoder doesn’t ignore small-but-important code features).

**Table S2.** Ablation of Auxiliary Objectives on Discriminative Tasks

| **Model Variant** | **AUROC (HF)** | **AUPRC (HF)** | **AUROC (T2DM )** | **AUPRC (T2DM )** |
| --- | --- | --- | --- | --- |
| Full (MFP + NTP) | 0.923 | 0.710 | 0.817 | 0.707 |
| – NTP only | 0.893 | 0.674 | 0.795 | 0.682 |
| – MFP only | 0.915 | 0.697 | 0.803 | 0.695 |
| – MFP & NTP | 0.870 | 0.640 | 0.780 | 0.660 |

Concurrently training on MFP and NTP, alongside the main language modeling objective, clearly forced the model to attend the structured data in a way that pure language modeling would not. This is evident from the baseline comparisons: models like CEHR-BERT and CLMBR also leverage sequential structure (CEHR-BERT via a Transformer over code sequences, CLMBR via next-code prediction). Yet GDP’s joint training with a generative objective and explicit temporal self-supervision appears to instill a more integrated understanding. In essence, GDP’s pretraining strategy – generate text conditioned on EHR while predicting masked and next events – produced a multifaceted representation that benefited both types of tasks. Our results support this: GDP-Instruct outperformed a similarly-sized instruct-tuned LLMs that were not pretrained on structured EHR data (Table 2 and 3 baselines), indicating that grounding text generation in patient timelines (through cross-attention and auxiliary losses) yields more relevant and factual narratives than a generic medical LLM fine-tuned on the same summaries. Meanwhile, GDP-Non-Instruct’s substantial boost over baselines like FPM or CLMBR on predictive tasks demonstrates the value of combining language-model pretraining with temporal encoding. We note that CEHR-BERT and CLMBR were strong in their domains, but lacked a generative text pretraining component; GDP’s unique fusion of these elements likely explains its superior overall performance. These anecdotal observations are not rigorously quantified and are presented as preliminary exploration to motivate future work on clinical QA benchmarks, not as evidence of validated capability.

## Qualitative evaluation via interactive chat

Beyond static benchmarks, we explored GDP’s potential in an interactive EHR question-answering setting. We developed a prototype chat interface where a user (e.g. a clinician, or health informatics specialist) can query the model about a patient’s case after “loading” that patient’s EHR data into the model’s context. Using held-out test patient records, we observed how the two GDP variants responded to ad-hoc questions. For example, given a patient’s structured data and notes indicating an admission for pneumonia, we asked: “Q: What were the main events during this hospital stay?” GDP-Instruct responded with a concise summary: “The patient was admitted with a high fever and cough. They were started on IV antibiotics for pneumonia, required supplemental oxygen briefly, and improved over 3 days. Key events include a fever spike to 39.2°C on day 1, blood cultures growing Streptococcus pneumoniae, and transition from IV to oral antibiotics on day 4 before discharge.” In contrast, GDP-Non-Instruct produced a less organized answer: “Pneumonia. They got antibiotics. Fever went up (39.2). Blood culture positive for strep pneumo. O given.” Both are factually correct, but the instruct model’s answer was more fluent and provided a clearer narrative flow, likely reflecting its training to generate human-like summaries. We also asked follow-up questions such as “Q: What follow-up care is recommended?”. GDP-Instruct was able to infer and respond with a reasonable recommendation (e.g., “Follow-up with the primary care physician in 1 week, repeat chest X-ray in 4-6 weeks, and continue oral antibiotics for the prescribed course.”), whereas GDP-Non-Instruct tended to give more generic or hesitant answers (sometimes just restating to follow up with a doctor without specifics). These anecdotal tests underscore that instruction tuning imparts better conversational abilities – GDP-Instruct was adept at understanding the question and formulating a contextually appropriate answer using the EHR data, effectively acting like a clinical assistant. GDP-Non-Instruct, while containing the necessary information in its latent representation, was less able to articulate it without direct prompting. This qualitative use-case highlights a future application of models like GDP: interactive, EHR-aware decision support systems. It also shows the value of bridging generative and predictive capacity – the model not only predicts risks, but can explain and discuss a patient’s case in natural language. We note, however, that these chat observations were not rigorously quantified and are meant to inspire further evaluation on clinical QA benchmarks, which is the future plan.

## Evaluation Metrics Definitions

F1 is the harmonic mean of precision and recall, reflecting a balance between sensitivity and specificity.

$$Precision=\frac{TP}{TP+FP} (1)$$

$$Recall=\frac{TP}{TP+FN} (2)$$

$$F_{1}=2\times\frac{\text{Precision}\times\text{Recall}}{\text{Precision}+\text{Recall}} (3)$$

$$Accuracy=\frac{TP+TN}{TP+TN+FP+FN} (4)$$

$$AUROC=\int_{0}^{1} \text{TPR}\left( \text{FP}\text{R}^{\text{-1}}\left( t \right) \right) dt (5)$$

$$AUPRC=\int_{0}^{1} \text{Precision}\left( \text{Recall}^{-1}\left( r \right) \right) dr (6)$$

Where TP / TN/ FP / FN denote true positives, true negatives, false positives, and false negatives, respectively. TPR = TP/(TP + FN) and FPR = FP/(FP + TN).The integrals for AUROC and AUPRC are typically estimated via trapezoidal approximation over the ROC and precision–recall curves.

For generative performance, we used standard natural language generation (NLG) metrics on the test set of discharge summary. We computed ROUGE-1, ROUGE-2, and ROUGE-L F1-scores (covering unigram overlap, bigram overlap, and longest common subsequence overlap) between the generated summaries and reference summaries. We report the F-measure variant of ROUGE, which balances precision and recall of overlap. We also calculated BLEU scores (up to BLEU-4, cumulative) which measure n-gram precision – an indicator of how exactly the model reproduced the reference phrasing. However, since strict n-gram matches can be overly harsh for this task (there can be many ways to write the same clinical fact), we included BERTScore, which uses a pre-trained language model to assess semantic similarity between the generated and reference text. BERTScore outputs a similarity score in [0,1]. All these metrics were computed using standard libraries (rouge-score, nltk for BLEU, and the BERTScore package with the recommended biomedical BERT model). We considered the reference summaries written by clinicians as the gold standard, and we averaged the scores across all test samples for each model (Table 2). To test for significance in metric differences, we used paired bootstrap resampling (e.g., for ROUGE-L differences between GDP-Instruct and baseline LLM).

$$ROUGE\_N\_F1=2\times\frac{Overlap\_Precision\_N\times Overlap\_Recall\_N}{Overlap\_Precision\_N+Overlap\_Recall\_N} (7)$$

$$BLEU=BP\times\exp!\left( \sum_{n=1}^{4} w_{n} \ln p_{n} \right) (8)$$

$$BERTScore\_F1=2\times\frac{Precision\_BERT\times Recall\_BERT}{Precision\_BERT+Recall\_BERT} (9)$$

Where,

$$\text{Overlap}\text{\_}\text{Precisio}\text{n}_{\text{N}}=\frac{\sum\text{matched N}\text{‐}\text{grams}}{\sum\text{generated N}\text{‐}\text{grams}} (10)$$

$$\text{Overlap}\text{\_}\text{Recal}\text{l}_{\text{N}}=\frac{\sum\text{matched N}\text{‐}\text{grams}}{\sum\text{reference N}\text{‐}\text{grams}} (11)$$

$$p_{n}=\text{Overlap}\text{\_}\text{Precisio}\text{n}_{\text{n}} (12)$$

$$\text{Precisio}\text{n}_{\text{BERT}}=\frac{1}{L}\sum_{i=1}^{L} \max_{j} \text{sim}\left( t_{i},s_{j} \right) (13)$$

$$\text{Recal}\text{l}_{\text{BERT}}=\frac{1}{M}\sum_{j=1}^{M} \max_{i} \text{sim}\left( s_{j},t_{i} \right) (14)$$

BP = { 1, if c > r

exp(1 – r/c), if c ≤ r}
